# GLUT-1 expression is largely unrelated to both hypoxia and the Warburg phenotype in squamous cell carcinomas of the vulva

**DOI:** 10.1186/1471-2407-14-760

**Published:** 2014-10-12

**Authors:** Arnulf Mayer, Marcus Schmidt, Alexander Seeger, André Franke Serras, Peter Vaupel, Heinz Schmidberger

**Affiliations:** Department of Radiooncology and Radiotherapy, University Medical Center, Langenbeckstrasse 1, 55131 Mainz, Germany; Department of Obstetrics and Gynecology, University Medical Center, Mainz, Germany

**Keywords:** Vulvar carcinoma, Hypoxia, Glucose transporter, Carbonic anhydrase IX, Hexokinase 2, Pyruvate kinase M2, Warburg effect

## Abstract

**Background:**

Strongly increased uptake of glucose is a hallmark of solid malignant tumors. This phenotype can be triggered by hypoxia-induced gene expression changes or can occur independently of hypoxia as a consequence of malignant transformation itself, and is often referred to as the Warburg effect. The glycolytic phenotype has been associated with malignant progression and resistance to radio- and chemotherapy.

**Methods:**

We have chosen squamous cell carcinomas of the vulva (SCC-V) as a representative solid tumor entity to study the central players of this pathway, namely glucose transporter (GLUT)-1, carbonic anhydrase (CA) IX, hexokinase (HK)-2 and pyruvate kinase (PK)-M2, and have investigated their relationships to tumor microvessels (CD34, αSMA) and proliferation (Ki67). Expression of these proteins was analyzed in 38 SCC-Vs, 5 vulvar dysplasias and 10 non-neoplastic squamous epithelia of the vulva using multiparametric immunohistochemistry in registered serial sections (MIRSS).

**Results:**

Expression of GLUT-1 in invasive carcinomas was predominantly located in the outer layers of the tumor cell aggregates close to the vascularized tumor stroma, and only to a lesser extent colocalized with CA IX, which was repeatedly found at larger diffusion distances away from microvessels. CA IX expression was lower in invasive carcinomas compared to dysplasias and non-neoplastic tissue and higher in recurrent vs. primary tumors. Ki67-positive proliferating cells were partially colocalized with GLUT-1. However, HK-2 and PK-2 - proteins centrally involved in the Warburg phenotype - did not show such a correlation.

**Conclusions:**

Consistent with prior studies, the pattern of GLUT-1 clearly indicated that a large part of its expression is presumably unrelated to hypoxia. However, there was also no association with HK-2 and PK-M2, suggesting that the functional background of this expression is also independent of aerobic glycolysis. CA IX may be worth consideration as a marker of biological hypoxia, as should its pathophysiological consequences in SCC-V.

**Electronic supplementary material:**

The online version of this article (doi:10.1186/1471-2407-14-760) contains supplementary material, which is available to authorized users.

## Background

The glucose transporter (GLUT)-1 and carbonic anhydrase (CA) IX are upregulated by the transcription factor hypoxia-inducible factor (HIF)-1. Since more than 50% of solid malignant tumors contain hypoxic tissue areas [1], it is not surprising that these entities have a high prevalence of expression of these proteins. In addition, a prevailing school of thought, based on the pioneering work of Warburg on cancer metabolism [2] asserts that glucose uptake and aerobic glycolysis is higher in malignant cells than in their physiological counterparts, independent of hypoxia [3]. A direct pathophysiological link connects GLUT-1 to CA IX since increased uptake of glucose into the (tumor) cell, and its aerobic glycolysis is associated with an increased production of lactate and protons. Carbonic anhydrase IX is thought to play an important role in the elimination of these protons from the cytosol of cancer cells with the aim of preventing an acidification of their intracellular environment [4]. This model is supported by reports which have described a co-expression of GLUT-1 and CA IX *in vivo*. These include, e.g., the data presented by Rademakers et al. on squamous cell carcinoma of the head and neck [5], Airley et al. [6] on cervical cancer as well as our own recent communication on glioblastomas [7]. Due to the fact that a large number of clinical studies have identified correlations between the expression of GLUT-1 and CA IX and a poorer patient prognosis [8], many authors believe that the glycolytic phenotype is also causally involved in the pathogenesis, progression and therapeutic resistance of malignant disease (see [9] for an overview). Of importance is the finding of Sattler et al. [10], who have recently shown that increased concentrations of lactate and pyruvate - end products of aerobic glycolysis - strongly correlate with resistance to fractionated radiotherapy. Moreover, there is also significant evidence supporting glycolysis-mediated resistance to some forms of chemotherapy (e.g., [11]).

Squamous cell carcinomas of the vulva (SCC-V) are a relatively rare entity, but become much more prevalent with increasing patient age (17/100,000 women over 75 years, [12]). Patient prognosis is determined most strongly by the depth of tumor invasion into the underlying stroma and a proportional increase in the occurrence of lymph node metastases. Primary therapy traditionally consisted of radical surgery, but this strategy has largely been abandoned in favor of multimodal approaches which integrate less extensive surgery with neoadjuvant or adjuvant radiotherapy (±chemotherapy) in order to improve both oncologic results and patient quality of life.

In the present work, we have investigated central elements of putative hypoxic and glycolytic phenotypes in SCC-V, since they may be relevant for the efficacy of neoadjuvant/adjuvant treatment regimes. SCC-V have been reported to be hypoxic by us [13] and others [14], but we also considered hypoxia-independent glycolysis, as outlined above. We have employed multiparametric immunohistochemistry in registered serial sections (MIRSS), a novel technique developed in our laboratory [7], which allows correlation of multiple antigens. Using this method we have analyzed the spatial expression patterns of GLUT-1 and CA IX in relation to (i) each other, (ii) the CD34/αSMA-positive tumor microvasculature, (iii) Ki67-positive proliferating cells and (iv) two key proteins widely believed to be causally involved in the “Warburg phenotype” of hypoxia-independent, aerobic glycolysis of malignant cells, hexokinase (HK)-2 [15] and pyruvate kinase (PK)-M2 [16]. In addition to 38 invasive SCC-Vs, 5 vulvar dysplasias and 10 non-neoplastic tissue specimens of the vulva containing squamous epithelium were analyzed using the same methodology.

## Methods

### Tissue specimens

Histological sections of 38 invasive squamous cell carcinomas of the vulva, 5 vulvar intraepithelial neoplasias and 10 non-neoplastic specimens containing squamous epithelium were obtained from the archive of the Department of Obstetrics and Gynecology, University Medical Center, Mainz. The study has been approved by the local medical ethics committee (Ethikkommission der Landesärztekammer Rheinland-Pfalz, No. 837.287.05). Clinical data of these patients are listed in Additional file 1: Table S1.

### Immunohistochemistry

Serial sections of 3 μm were prepared from paraffin blocks using a high-precision microtome and dried overnight at 37°C (Histology core facility, University Medical Center, Mainz). On the next day, specimens were dewaxed in two changes of fresh xylene and then rehydrated in a descending alcohol series. Retrieval of antigenic binding sites was performed by heating specimens in appropriate buffers (see Table 1 for details) in a steamer (Braun FS 10, Braun, Kronberg, Germany) for 40 min. The primary antibodies and incubation conditions used are listed in Table 1. Biotin-free, micropolymer-based Vector Immpress reagents (Vector Laboratories, Burlingame, CA) were used for the detection and visualization of primary antibody binding. Reagents were applied overnight at 4°C. Negative control specimens were incubated in PBS without the primary antibody under the same conditions. Diaminobenzidine (DAB) was used as the peroxidase substrate. Slides were counterstained with Mayer’s hematoxylin, dehydrated in an ascending alcohol series, and covered with a coverslip using Roti-Histokitt mounting medium (Carl Roth, Karlsruhe, Germany). Digital images of the specimens were acquired using a histology scanner (OpticLab H850, Plustek, Taipei, Taiwan). For the analysis of the mean distance to the nearest microvessel of GLUT-1 and CA IX positive cells in invasive carcinomas, all CD34, GLUT-1 and CA IX slides of these specimens were additionally scanned with Leica/Aperio and Hamamatsu slide scanners at 20x/40x magnification. Only immunostaining compatible with the known biological function and corresponding subcellular localization of each antigen was considered as being evaluable as marker expression (see Table 1).

**Table 1:** **Antibodies (AB), immunohistochemical techniques and resulting staining patterns**

Antigen	Epitope retrieval buffer	Primary AB (Cat.-No., dilution)	Primary AB supplier	Detection system	Staining pattern
**GLUT-1**	Citrate, pH 6.0	Cat.-No. GI817C01 (poly**), 1:400	DCS, Hamburg, Germany	ImmPRESS Anti-Rabbit (Vector)	Membranous
**CA IX**	Citrate, pH 6.0	Cat.-No. 3829–1 (mono*), 1:400	Epitomics, Burlingame, CA	ImmPRESS Anti-Rabbit (Vector)	Membranous
**CD34 Class II**	Tris/EDTA pH 9.0	Cat.-No. M7165 (mono*), 1:200	DAKO, Hamburg, Germany	ImmPRESS Anti-Mouse (Vector)	Membranous
**Ki67**	Citrate, pH 6.0	Cat.-No. ab16667 (mono*), 1:2000	Abcam, Cambridge, UK	ImmPRESS Anti-Rabbit (Vector)	Nuclear
**aSMA (Klon IA4)**	Citrate, pH 6.0	Cat.-No. A1922C002 (mono*), 1:200	DCS, Hamburg, Germany	ImmPRESS Anti-Mouse (Vector)	Cytoplasmic
**Hexokinase2 (C64G5)**	Citrate, pH 6.0	Cat.-No. 2867 (mono*), 1:200	Cell Signaling Technology, Danvers, MA	ImmPRESS Anti-Rabbit (Vector)	Cytoplasmic
**PKM2**	Citrate, pH 6.0	Cat.-No. 3198 (poly**), 1:100	Cell Signaling Technology, Danvers, MA	ImmPRESS Anti-Rabbit (Vector)	Cytoplasmic

*monoclonal antibody, **polyclonal antibody.

### Image preparation and registration

To enable spatial correlations, digital images of the scanned immunostains of GLUT-1 and CA IX (OpticLab scans) were registered to the corresponding Ki67 image. To achieve this, each source image (e.g., GLUT-1) and the corresponding target image (Ki67) were opened simultaneously in ImageJ on a 30 inch TFT monitor. Using the “multipoint tool”, approximately 20 corresponding landmarks were set in each image. The point selection was then converted to a “region of interest” (ROI) and saved to disk for later reference. Using the plugin “bunwarpj” [17] both images were registered using the following parameters: registration mode: “mono”; initial deformation: “fine”, final deformation: “super fine”, landmark weight =1, image weight =0.5, Verbose = yes. All other parameters were left at their default settings. Using this method, registration results showed geometric correspondence down to the level of relevant microstructures. For the analysis of the mean distance to the nearest microvessel in virtual slides (Leica and Hamamatsu scans), 20x/40x images were first reduced to a size which retained sufficient resolution for this type of analysis while being small enough (typically between 50 and 150 Megabyte per LZW-compressed TIFF image) to enable their processing in ImageJ [18]. This step was carried out in Image Pro Premier (IPP, Media Cybernetics, Rockville, MD). Images were then registered in a similar fashion as described above. However, since the distance analysis is critically dependent upon the utmost achievable precision of the registration process, we typically used 100 landmarks for each image (200 for each pair). CD34 specimens were used as the target images for both GLUT-1 and CA IX. Since (in our hands) the “bunwarpj” plugin (used for the slightly smaller OpticLab scans) was not able to register some of the larger images in this series, we used the ImageJ plugin “Landmark correspondences” instead [19], which consistently showed very good results with these large images. Representative examples of the registration results are shown in Additional file 2: Figure S1.

### Quantification of marker expression (OpticLab scans)

**Figure 1:**
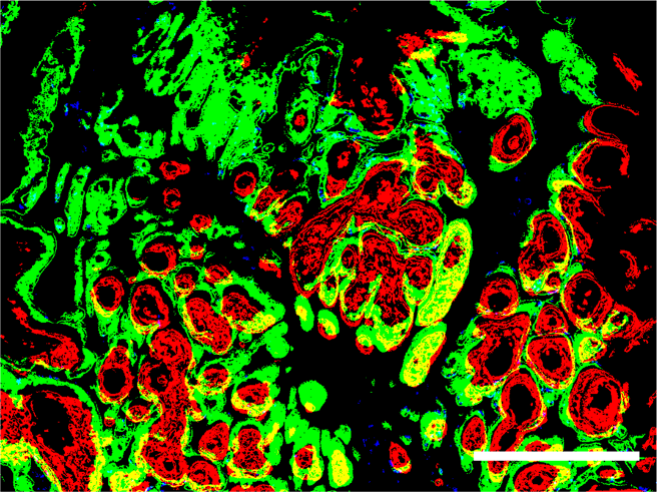
**Detail from a merged image of three registered entire tumor sections stained for GLUT-1, CA IX and Ki67.** Thresholded positive pixels are shown in green, red and blue, respectively. Overlap between GLUT-1 and CA IX is shown in yellow, pixels positive for GLUT-1 and Ki67 are shown in cyan. White bar =1 mm.

The extent of the expression of GLUT-1, CA IX, and Ki67 was quantified as the positive fraction of the total evaluable tumor area (of the entire histological section). Marker positive pixels were highlighted by color thresholding in Adobe Photoshop CS5 using the “wand tool”, while simultaneously examining the corresponding glass slides under the microscope to avoid the inclusion of regions that did not correspond to cancer cells. This was especially important in the case of GLUT-1, which is not only found in cancer cells but also exhibits a physiological expression in the cell membrane of red blood cells. Pixels thus labeled as positive for GLUT-1, CA IX and Ki67 were stored in the green, red and blue channels, respectively, of a new RGB (“merge”) image, which had the same pixel dimensions as the corresponding original digitized tumor specimens (Figure 1). To define the pixel size of the total tumor surface area (as opposed to the total surface area of the entire digitized specimen, which included various amounts of surrounding normal tissue), the section stained for CA IX was used as a representative reference. Here, the tumor area was marked manually in Adobe Photoshop CS5 using the lasso tool and subsequently converted to a black and white mask image which was saved to a file. The pixel dimensions of the tumor area were then quantified in ImageJ. For measurements of the percentage of positive tumor area for GLUT-1, CA IX and Ki 67, the number of positive pixels belonging to each of these antigens was determined using ImageJ’s "Color inspector 3D" plugin and related to the total tumor area. The extent of colocalization between GLUT-1 and CA IX was calculated as the percentage of colocalized pixels relative to the sum of the pixels of both antigens. To quantify the relationship of the expression of the aforementioned markers with proliferation, we calculated how many Ki67-positive pixels were simultaneously positive for GLUT-1 alone, both for GLUT-1 and CA IX or CA IX alone.

### Quantification of the mean distance to the nearest microvessel of GLUT-1 and CA IX in invasive carcinomas (Aperio/Hamamatsu scans)

Registered CD34, GLUT-1 and CA IX sections were converted to an image stack using ImageJ. This stack was imported into IPP and the tumor area was outlined as a region of interest (ROI). Further steps were carried out in this ROI only. CD34 positive microvessels, GLUT-1 and CA IX positive pixels were identified by thresholding in the HSL (hue, saturation, luminance) color space and converted to black and white masks. The CD34 mask image was converted to a 32 bit euclidean distance transformation, in which each grey value corresponds to the distance (in pixels) to the nearest microvessel. Grey values were consecutively converted to a calibrated distance (in μm). GLUT-1 and CA IX mask images and the distance transformation image were once again imported into ImageJ. GLUT-1 and CA IX positive pixels were turned into pixel “selections” which then were redirected to the distance transformation image to obtain the mean calibrated distance of the expression of each marker relative to the nearest microvessel.

### Statistical analysis

All statistical tests were performed using the SPSS software package (Version 21, IBM, Armonk, NY). The significance level was set at α=5% for all comparisons. Linear correlations between two variables were described by Spearman's rank correlation coefficient (ρ). Two-sided Mann–Whitney U tests were used for comparison of categorized variables. A two-sided Wilcoxon test was used for the analysis of the distances of GLUT-1 and CA IX (paired samples) relative to CD34 positive blood vessels.

## Results

### Visual assessment of the expression patterns

**Figure 2:**
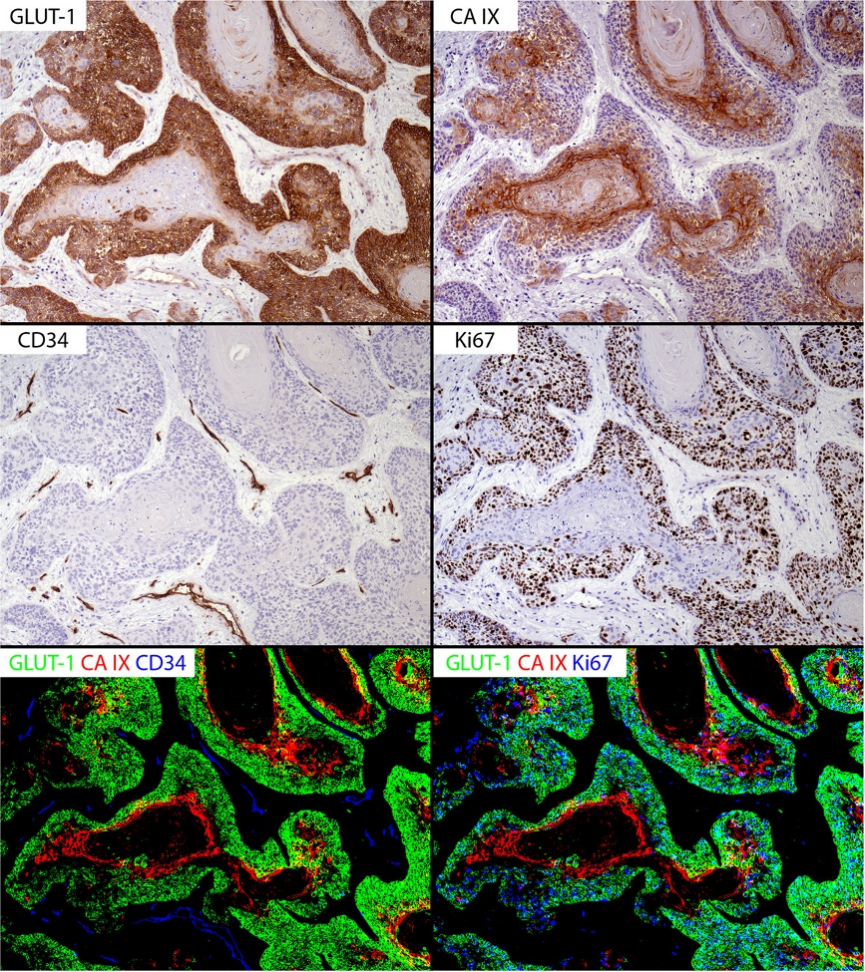
**Representative colocalization patterns of GLUT-1, CA IX, CD34 and Ki67 in an invasive squamous cell carcinoma of the vulva.** GLUT-1 (upper left panel) is expressed preferentially in the outer layers of the tumor cell aggregates, while CA IX (upper right panel) is expressed at a distance from the stroma, which contains CD34-positive tumor microvessels (middle left panel). The lower left panel shows a merged image of thresholded positive pixels of GLUT-1 (green), CA IX (red) and CD34 (blue) and illustrates minimal overlap (yellow) between GLUT-1 and CA IX. It also underscores the aforementioned spatial relationships of GLUT-1 and CA IX with CD34-positive microvessels (blue). Ki67-positive proliferating cells (middle right panel) are found in the same compartment as GLUT-1 positive cells. Conversely, the expression of Ki67 and CA IX is virtually mutually exclusive. The lower right panel shows a merged image of thresholded positive pixels of GLUT-1 (green), CA IX (red) and Ki67 (blue). Any overlap between Ki67 and GLUT-1 and Ki67 and CA IX is visible in cyan and pink colors, respectively. All panels: cropped frames from 10x magnification digital images.

In a first step, the expression patterns of all antigens were evaluated visually. In addition to viewing the slides with a light microscope at 10x or 20x magnification to assess details, low magnification views of sets of 4 complete digital tumor specimens, each stained for a different antigen and arranged in the same spatial orientation as the others were viewed side by side on a 30 inch monitor. GLUT-1 and CA IX were expressed in all specimens, but the extent of this expression varied considerably. In all non-neoplastic tissues and in most (i.e., 4 of 5) of the dysplasias, GLUT-1 was expressed most strongly in the cell layers immediately adjacent to the stroma and its intensity decreased and ultimately disappeared with increasing distance from the stromal compartment. Conversely, in both non-neoplastic tissues and dysplasias, the expression of CA IX persistently showed an opposite pattern with an expression intensity which increased with the cells’ distance from the stroma. Overlap of both proteins occurred in a zone between the aforementioned hotspots, but the extent of this zone was typically quite limited. Expression of GLUT-1 in non-neoplastic tissues and dysplasias colocalized with Ki67 positive (proliferating) cells, which, as expected, were located in the basal layer(s) of the squamous epithelia. Interestingly, a similar spatial distribution of the three antigens was also observed as the predominant pattern in the invasive carcinomas (Figure 2). Since a distant, “hypoxia-inducible” expression of GLUT-1 may be expected for this HIF-1 target gene, we subsequently refer to this GLUT-1 pattern as the “inverse” type, whereas the CA IX pattern was considered “typical” for a hypoxia-regulated protein (see discussion). The relationship between CD34, GLUT-1, CA IX and Ki67 was further characterized in quantitative analyses (see below).

HK-2 and PK-M2 showed a weaker and more diffuse expression pattern compared to the aforementioned antigens (Additional file 3: Figure S2). Since there was only negligible variation in the expression intensity of these antigens, quantitative colocalization analyses, e.g., with GLUT-1 and CA IX, would not have yielded additional information and have thus been omitted. It was evident from visual inspection alone that correlations between either HK-2 or PK-M2 with GLUT-1 or CA IX were definitely lacking.

As anticipated, the endothelial marker CD34 and smooth muscle actin (αSMA) exhibited a strong spatial association in and around intratumoral microvessels, with αSMA positive cells surrounding CD34-positive endothelial cells, in keeping with the known expression of αSMA in pericytes and vascular smooth muscle cells. Hence, α-SMA served as a biological plausibility control for the detection of CD34 positive microvessels. In addition, an independent subset of αSMA-positive cells showing no relationship to microvessels was observed in varying proportions. Pertaining to the phenotype of these cells and in accordance with the literature, these cells most probably represent (activated) myofibroblasts.

### Quantitative evaluation of the registered serial sections

#### Antigen-positive area fractions

**Figure 3:**
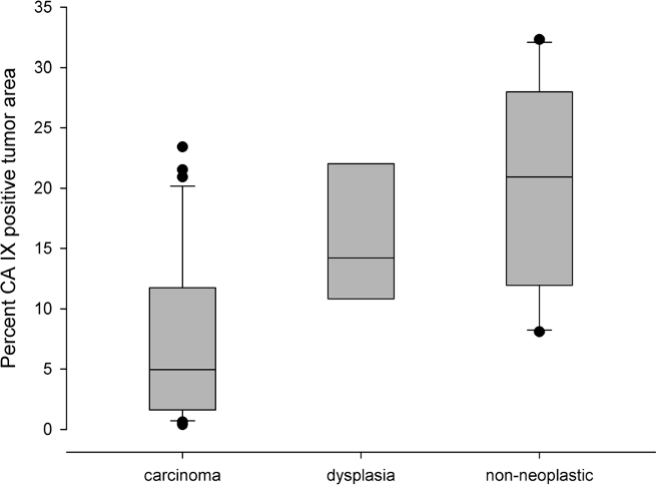
**Box and whisker plots illustrating the percentages of pixels positive for CA IX relative to the total tumor area in invasive carcinomas, dysplasias and non-neoplastic tissue.**

In a first quantitative analysis, we tested whether GLUT-1-, CA IX-, and KI 67-positive fractions of the entire tumor areas were distributed differently in invasive tumors, dysplasias and non-neoplastic squamous epithelium. Surprisingly, the non-neoplastic tissue specimens showed a significantly higher CA IX expression compared to the dysplasias (p =0.026) and in particular, to the invasive carcinomas (p =0.00005, Figure 3, Additional file 1: Tables S2–S4). Dysplasias also showed a higher proliferation compared to invasive tumors (p =0.026). In invasive carcinomas, the expression of GLUT-1 was much more pronounced than that of CA IX (quantified as the ratio of GLUT-1 positive pixels to CA IX positive pixels, which had a median value of 6.5), whereas this inequality was significantly less pronounced in dysplasias (p =0.018) and non-neoplastic tissues (p =0.000004), matching the lower overall expression of CA IX in invasive carcinomas (see also Additional file 1: Table S2).

#### Comparison of the expression patterns of GLUT-1 and CA IX

**Figure 4:**
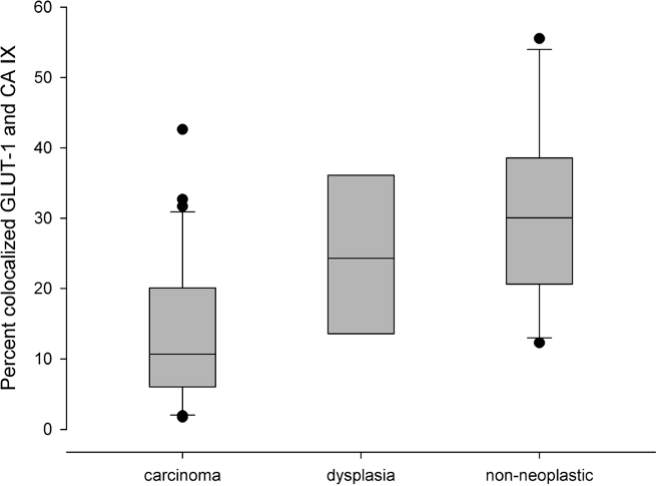
**Box and whisker plots illustrating the percentages of pixels positive for both GLUT-1 and CA IX relative to all GLUT-1- and CA IX-positive pixels in invasive carcinomas, dysplasias and non-neoplastic tissue.**

The visual impression of low colocalization of GLUT-1 and CA IX was confirmed in the quantitative analysis. For this purpose, the percentage of colocalized pixels relative to the sum of GLUT-1 and CA IX positive pixels was determined. In the invasive tumors, the colocalized fraction had a median value of only 10.7%. On the one hand, this value is very low in absolute terms and on the other hand it is significantly lower than in the dysplasias (p =0.023) and non-neoplastic tissues (p =0.0002, Figure 4).

#### Quantitative analysis of the mean distance of GLUT-1 and CA IX to the nearest microvessel

A comprehensive analysis of the distances of GLUT-1 and CA IX positive pixels relative to the nearest microvessels using a distance transformation image of the CD34 microvessel “map” revealed a mean distance of 85 μm (SD 25 μm) for GLUT-1 and 124 μm (SD 32 μm) for CA IX. This difference was highly significant (p <0.0001). When looking at individual specimens, not even a single instance was found in which the mean distance was larger for GLUT-1 than for CA IX. These findings underscore our interpretation of an “inverse” expression pattern of GLUT-1 and a “typical”, hypoxia-related pattern of CA IX.

#### Expression of GLUT-1 and CA IX in relation to proliferation (Ki67)

**Figure 5:**
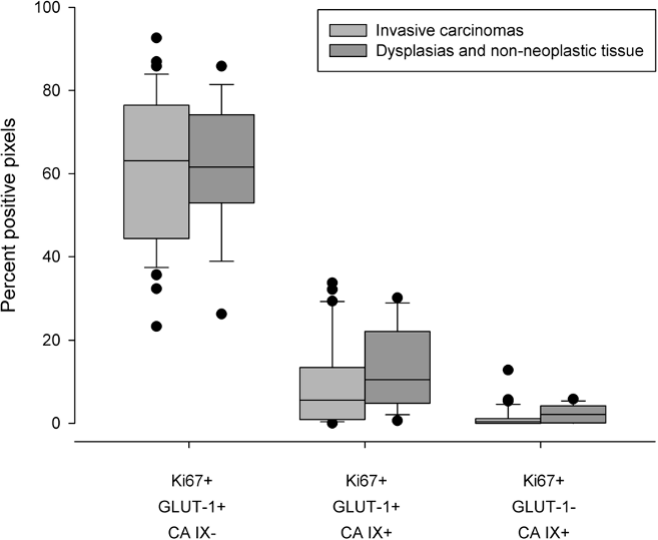
**Grouped Box and whisker plots illustrating the percentages of pixels positive for Ki67 and GLUT-1, CA IX or both proteins grouped by invasive carcinomas (light grey) and dysplasias/non-neoplastic tissue (dark grey).**

In all three types of tissue, Ki67-positive pixels were also positive for GLUT-1 (but not for CA IX) in a high percentage, positive for both GLUT-1 and CA IX to a lesser degree, and positive for CA IX (but not GLUT-1) only on rare occasions (p <0.0000001 for both comparisons to the first group, see Figure 5).

#### Differences between primary and recurrent tumors

**Figure 6:**
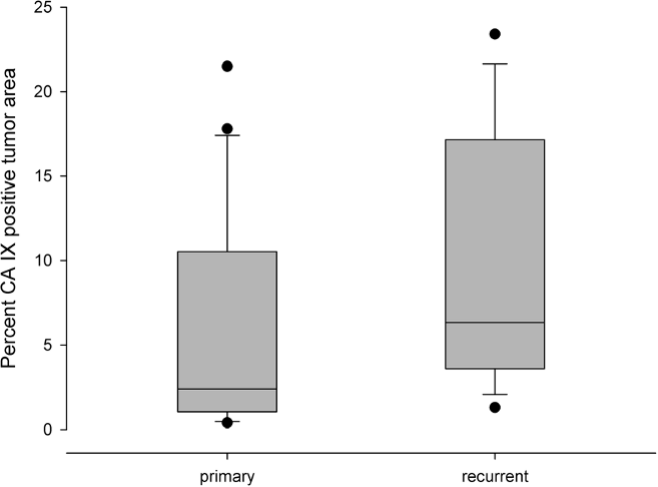
**Box and whisker plots illustrating the percentages of pixels positive for CA IX relative to the total tumor area in primary vs. recurrent invasive carcinomas.**

The recurrent tumors (n =16) had a significantly higher expression (p =0.042) of CA IX as compared to the primary tumors (n =22); see Figure 6. In addition, the percentage of Ki67-positive pixels that were also positive for both GLUT-1 and CA IX were higher in the recurrent tumors (p =0.048).

## Discussion

From the present study we have obtained novel and partially unexpected insights into the pathophysiological ramifications of the expression of the hypoxia-associated markers GLUT-1 and CA IX in squamous cell carcinomas of the vulva. Using polarographic needle electrodes, studies by Stone et al. [14] and Vaupel et al. [13] have described profound hypoxia in squamous cell carcinomas of the vulva. However, we [20, 21] and others [22, 23] have also reported earlier that the expression intensity of GLUT-1 and CA IX is not directly proportional to microregional hypoxia, using the same method. Consistent with these latter data, the present study describes a largely inverse, i.e., hypoxia-independent expression pattern of GLUT-1 and a diminished expression of CA IX in invasive tumors compared to dysplasias and non-malignant vulvar squamous epithelium. On a descriptive level, similar observations have been made in a prior study [24], but these authors did not carry out quantitative analyses. Indeed, we observed a median 6.5-fold higher expression of GLUT-1 compared to CA IX in the invasive tumors, whereas the preponderance of GLUT-1 in dysplasias and non-neoplastic tissues was significantly less pronounced. Constitutive expression of GLUT-1 has been reported, e.g., in red blood cells, normal capillaries of the brain and in the parathyroid gland, while expression of CA IX is typically found in the normal epithelia of stomach and gallbladder [25]. Expression of these proteins has also been described in normal vulvar tissue and vulvar dysplasias in earlier studies, but was found to be weaker compared to the invasive lesions [26, 27]. Disparate findings between these and our communications may in part relate to the fact that we have performed quantitative analyses on complete surgical specimens while the previous investigators carried out semiquantitative analyses in biopsy specimens [26] or tissue microarrays [27]. Nevertheless, the finding of a reduced CA IX expression in invasive lesions in our study is surprising. Although one of the aforementioned studies which have directly measured the oxygenation of vulvar carcinomas [13] included measurement of normal tissue, these data were obtained in the mons pubis, not in normal healthy vulvar tissue. Therefore, it cannot be excluded that normal vulvar epithelium indeed contains hypoxic areas which have not yet been recognized. Owing to the highly diffuse infiltrative nature of vulvar carcinoma, which is an important factor regarding the unfavorable clinical tendency of the disease to recur, better oxygenation of invasive tumors compared to the corresponding normal tissues seems conceivable due to the fact that the tumor infiltrates into a highly vascular connective tissue. The finding of a higher CA IX expression in our study in recurrent tumors is consistent with this idea, since vascularity and the resulting oxygen transport capacity of connective tissue are often compromised after previous surgery or radiotherapy.

Our data further indicate a preferential colocalization of Ki67 with GLUT-1, but not with CA IX in invasive carcinomas, similar to the findings in dysplastic lesions and non-neoplastic tissues. These data are consistent with the largely inverse expression of GLUT-1 in the outer layers of the cancer cell aggregates and the expression of CA IX in tumor areas at a greater distance from neighboring microvessels. Hence, CA IX positive subvolumes are presumably poorly perfused and represent hypoxic tissue areas. The inhibitory effect of chronic hypoxia on cell proliferation has long been recognized [28]. At first glance, these data might suggest the conjecture that a hypoxia-independent glycolytic phenotype associated with cell proliferation, compatible with Warburg's original hypothesis, is present in SCC-V. However, we could not establish evidence supporting such an interpretation. Both HK-2 and PK-M2 are considered to be of central functional importance for aerobic glycolysis [15, 16], yet their expression patterns lacked any clear association with either GLUT-1 or CA IX in our study. The possibility, however, that different results might be obtained with other antibody clones, cannot be completely ruled out. Our results should, therefore, be regarded as preliminary at this point in time. Nevertheless, under conditions of a highly active glycolytic metabolism with resultant accumulation of pyruvate and lactate (Warburg phenotype), induction of HIF-1α would be expected to occur in the GLUT-1 positive tumor areas according to the findings of Lu et al. [29], which should then, in turn, transactivate CA IX in the same tumor microregions, since CA IX is known to be one of the most robust and strongly induced target genes of HIF-1 [30, 31]. Hence, doubts regarding the equivalence of GLUT-1 staining with an active “Warburg-type” metabolism in our study may in fact be valid.

The expression pattern of GLUT-1, as described in the present study, may neither be related to hypoxia nor to “aerobic glycolysis”, i.e., the Warburg effect. The increased glucose uptake capacity of GLUT-1 positive cells near the tumor stroma may have a different purpose, which is independent of energy metabolism. It is conceivable, e.g., that a high glucose uptake is necessary to provide ribose-5-phosphate for DNA synthesis, which is supplied by reactions of the pentose phosphate cycle. The latter pathway is also important for the regeneration of reduced glutathione, which may be crucial for the protection of the integrity of the DNA of proliferating cells against oxygen radicals. Finally, glyceraldehyde-3-phosphate, a glycolytic intermediate, is a building block for phospholipids and triacylglycerols, which are essential for the assembly of the cell membranes of newly generated cells.

## Conclusions

It can be stated that the expression of GLUT-1 in vulvar cancers is significantly different from the pattern found in some other tumor entities, and may be derived from physiological characteristics of the proliferative compartment of the vulvar squamous epithelium and commandeered by the neoplastic cells derived from it. The pressing question as to whether the loss of hypoxia-independent expression of GLUT-1 in subregions of vulvar carcinomas is equivalent to dedifferentiation and may be associated with a poorer patient prognosis should be answered in a future study with a significantly higher number of patients. Conversely, the expression pattern of CA IX is compatible with a marker function for local hypoxia, although probably not on an absolute scale, as has been demonstrated in cancers of the uterine cervix [20]. Reduction of CA IX in vulvar carcinomas may indicate that the invasion of these vulvar cancer cells may be in part driven by hypoxia, a possibility that warrants investigation in further experimental, translational and clinical studies.

## Electronic supplementary material

Additional file 1: Tables S1–S4: These tables contain clinical/histopathological data (Additional file 1: Table S1) and details regarding the antigen-positive tumor areas in invasive carcinomas, dysplasias and non-neoplastic tissue of the vulva (Additional file 1: Tables S1–S4). (DOCX 22 KB)

Additional file 2: Figure S1: Details from the registration of GLUT-1 and CA IX to CD34 using the Aperio and Hamamatsu scans. Each row represents a different tumor. (JPEG 6 MB)

Additional file 3: Figure S2: Staining patterns of GLUT-1 (upper left panel), CA IX (lower left panel), Hexokinase-2 (HK-2, upper right panel) and pyruvate kinase type M2 (PK-M2, lower right panel). GLUT-1 and CA IX both show a clearly demarcated and strong signal which is unequivocally restricted to a subtype of the cells present in the tissue slice. Conversely, HK-2 and PK-M2 staining is weak and more diffusely distributed throughout the tumor section. Images show the same subregion of the tumor but have not been registered using the described methodology. (JPEG 5 MB)
