# Supplementary material for: GLUT-1 expression is largely unrelated to both hypoxia and the Warburg phenotype in squamous cell carcinomas of the vulva
Source: BMC Cancer. 2014 Oct 12;14:760. doi: 10.1186/1471-2407-14-760 (PMC4210616; doi:10.1186/1471-2407-14-760)
Supplement: Supplementary file 1 — Additional file 1: Tables S1–S4: These tables contain clinical/histopathological data (Additional file 1: Table S1) and details regarding the antigen-positive tumor areas in invasive carcinomas, dysplasias and non-neoplastic tissue of the vulva (Additional file 1: Tables S1–S4). (DOCX 22 KB) [file 12885_2014_4944_MOESM1_ESM.docx]

**Additional Table 1. Clinical data for 38 patients with invasive squamous cell carcinomas of the vulva**

| **Patient No.** | **Primary tumor vs. recurrence** | **TNM stage** | **Grading** |
| --- | --- | --- | --- |
| 1 | primary | pT2, N1, Mx | 1 |
| 2 | recurrence | rpT3, pN1, Mx | 2 |
| 3 | recurrence | rpTx, rpN1, cM0 | 2 |
| 4 | primary | pT2, N1, Mx | 2 |
| 5 | primary | pT2, pN0, Mx | 2 |
| 6 | primary | pT2, pN1, Mx | 1 |
| 7 | recurrence | rpT2, pNx, Mx | 2 |
| 8 | primary | pT2, pN2, Mx | 1 |
| 9 | primary | pT1b, pN1, Mx | 2 |
| 10 | recurrence | rpT2, pNx, Mx | 2 |
| 11 | primary | pTx, pNx, Mx | n.a. |
| 12 | primary | pT2, pN2, Mx | 2 |
| 13 | recurrence | rpT1b, pN0, Mx | 2 |
| 14 | primary | pT2, pN0, Mx | 1 |
| 15 | recurrence | rpT1b, cN0, Mx | 2 |
| 16 | primary | pT1b, pN0, Mx | 2 |
| 17 | recurrence | rpT3, pNx, Mx | 2 |
| 18 | primary | pT1b, pN0, Mx | 3 |
| 19 | primary | pT2, pN0, Mx | 3 |
| 20 | primary | pT2, pN2, Mx | 3 |
| 21 | recurrence | rpT2, cNx, Mx | 2 |
| 22 | primary | pT1, pN0, pMx | 1 |
| 23 | recurrence | pT3, pN1, pMx | n.a. |
| 24 | recurrence | pT3, pN1, cM0 | n.a. |
| 25 | recurrence | ypT2, cNx, Mx | 2 |
| 26 | primary | pT2, pN1, Mx | 2 |
| 27 | primary | pT2, pN0, Mx | 2 |
| 28 | recurrence | pT2, pN1, Mx | 3 |
| 29 | recurrence | pT2, pN1, cM0 | 3 |
| 30 | primary | pT1b, pN0, Mx | 2 |
| 31 | primary | pT1b, pNx, Mx | 1 |
| 32 | primary | pT1b, pN0, Mx | 1 |
| 33 | primary | pT2, pN1, Mx | 2 |
| 34 | recurrence | pT2, pN0, Mx | 2 |
| 35 | recurrence | rpT1b, pNx, Mx | 2 |
| 36 | recurrence | rpT1b, pN0, Mx | 2 |
| 37 | primary | pT1a, pN0, Mx | 2 |
| 38 | primary | pTx, pNx, Mx | 2 |

n.a. = not available

**Additional Table 2. Antigen-positive tumor areas in 38 invasive squamous cell carcinomas of the vulva**

| Patient No. | % GLUT positive tumor area | % CA IX positive tumor area | % Ki67 positive tumor area |
| --- | --- | --- | --- |
| 1 | 15.8 | 5.3 | 4.8 |
| 2 | 46 | 4 | 0.3 |
| 3 | 19.5 | 20.9 | 3.4 |
| 4 | 44.3 | 17.8 | 16.4 |
| 5 | 53.5 | 1.3 | 3.3 |
| 6 | 36.5 | 1.9 | 1.8 |
| 7 | 32 | 1.3 | 3.1 |
| 8 | 58.3 | 16.5 | 1.4 |
| 9 | 35.2 | 4.9 | 8.5 |
| 10 | 24.2 | 3.6 | 0.6 |
| 11 | 7.2 | 0.6 | 8.9 |
| 12 | 58 | 5.8 | 1.5 |
| 13 | 30.4 | 11.3 | 14.7 |
| 14 | 7 | 0.4 | 4.3 |
| 15 | 52.5 | 9.9 | 1.9 |
| 16 | 6.9 | 2.3 | 0.5 |
| 17 | 21.6 | 2.4 | 0.4 |
| 18 | 19.4 | 1.1 | 0.9 |
| 19 | 28.4 | 13 | 2.3 |
| 20 | 9.9 | 0.4 | 0.6 |
| 21 | 21.4 | 5.8 | 6.3 |
| 22 | 13 | 9.7 | 0.9 |
| 23 | 30.4 | 5 | 4.4 |
| 24 | 26.3 | 3.6 | 0.6 |
| 25 | 71.9 | 6.9 | 5.7 |
| 26 | 19.2 | 0.9 | 0.9 |
| 27 | 12.9 | 1.7 | 3.1 |
| 28 | 36 | 23.4 | 1.8 |
| 29 | 66.2 | 20.1 | 0.5 |
| 30 | 41.9 | 6.7 | 3.3 |
| 31 | 9.4 | 0.7 | 0.7 |
| 32 | 56 | 16.3 | 1.6 |
| 33 | 65.8 | 21.5 | 39.4 |
| 34 | 20.7 | 7.3 | 17.1 |
| 35 | 14.4 | 2.5 | 0.7 |
| 36 | 27.1 | 19.1 | 1 |
| 37 | 20.2 | 2.5 | 0.9 |
| 38 | 48.4 | 1.2 | 2.2 |
|  |  |  |  |
| **Mean** | 31.8 | 7.4 | 4.5 |
| **Median** | 27.8 | 5.0 | 1.9 |

**Additional Table 3. Antigen-positive areas in 5 dysplasias of the vulva**

| Patient No. | % GLUT positive tumor area | % CA IX positive tumor area | % Ki67 positive tumor area |
| --- | --- | --- | --- |
| 39 | 20.4 | 12.5 | 3.1 |
| 40 | 37.8 | 14.6 | 18.9 |
| 41 | 36.3 | 14.2 | 8.5 |
| 42 | 55.3 | 9.1 | 12.6 |
| 43 | 25.1 | 29.4 | 3.5 |
|  |  |  |  |
| **Mean** | 35.0 | 16.0 | 9.3 |
| **Median** | 36.3 | 14.2 | 8.5 |

**Additional Table 4. Antigen-positive areas in 10 non-neoplastic tissues of the vulva**

| Patient No. | % GLUT positive tumor area | % CA IX positive tumor area | % Ki67 positive tumor area |
| --- | --- | --- | --- |
| 44 | 23.8 | 12.8 | .9 |
| 45 | 47.8 | 27.3 | 1.2 |
| 46 | 21.3 | 8.1 | 5.9 |
| 47 | 54.5 | 22.0 | 5.7 |
| 48 | 32.6 | 19.8 | 3.2 |
| 49 | 41.2 | 30.0 | 1.8 |
| **50** | 18.5 | 20.3 | 3.2 |
| **51** | 57.8 | 32.3 | 3.0 |
| **52** | 32.7 | 9.3 | 9.7 |
| **53** | 28.2 | 21.5 | 1.2 |
|  |  |  |  |
| **Mean** | 35.8 | 20.3 | 3.6 |
| **Median** | 32.7 | 20.9 | 3.1 |
