# Supplementary figures and images for: GLUT-1 expression is largely unrelated to both hypoxia and the Warburg phenotype in squamous cell carcinomas of the vulva
Source: BMC Cancer. 2014 Oct 12;14:760. doi: 10.1186/1471-2407-14-760 (PMC4210616; doi:10.1186/1471-2407-14-760)

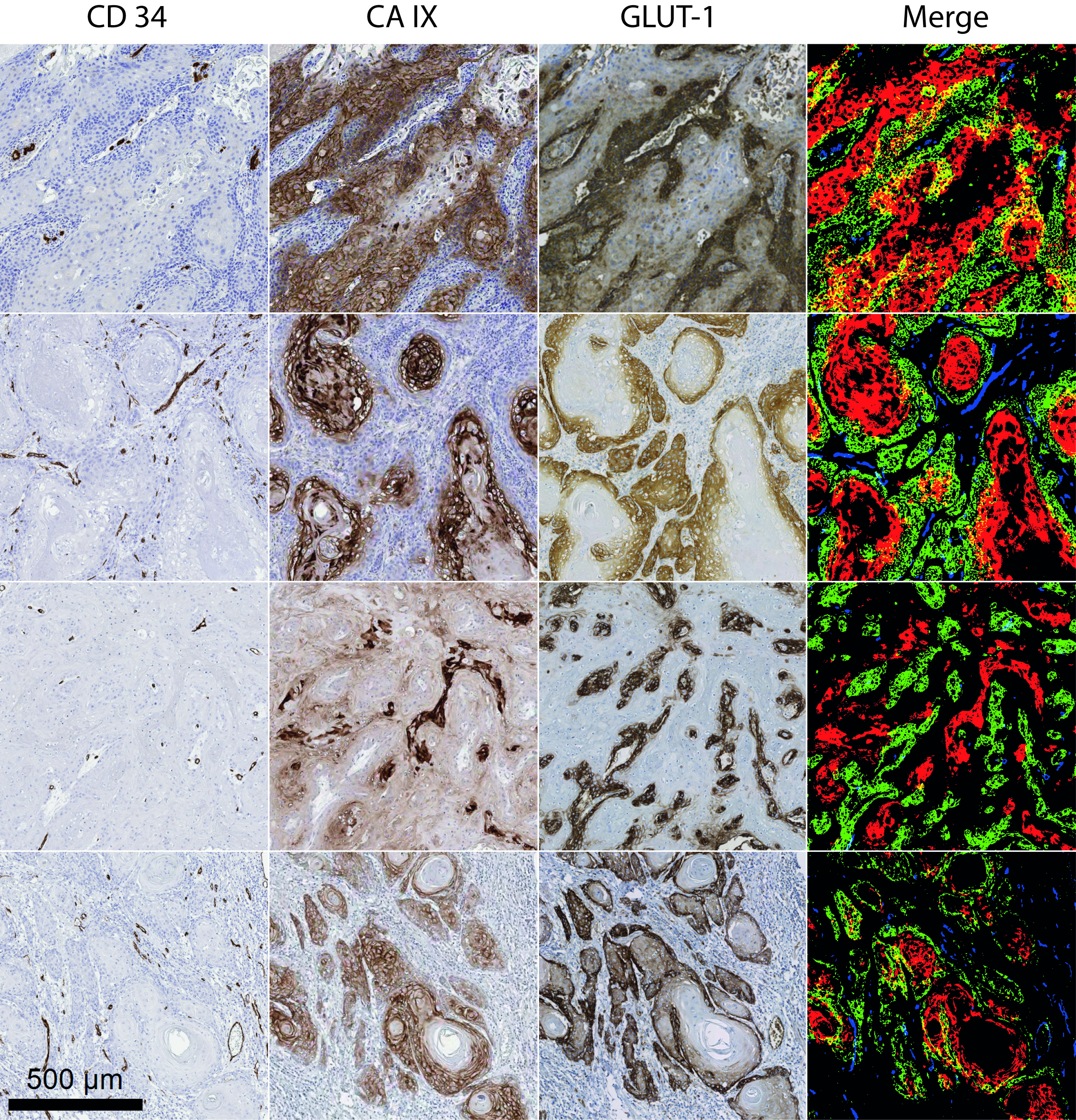

Supplement: Supplementary file 2 — Additional file 2: Figure S1: Details from the registration of GLUT-1 and CA IX to CD34 using the Aperio and Hamamatsu scans. Each row represents a different tumor. (JPEG 6 MB) [file 12885_2014_4944_MOESM2_ESM.jpeg]

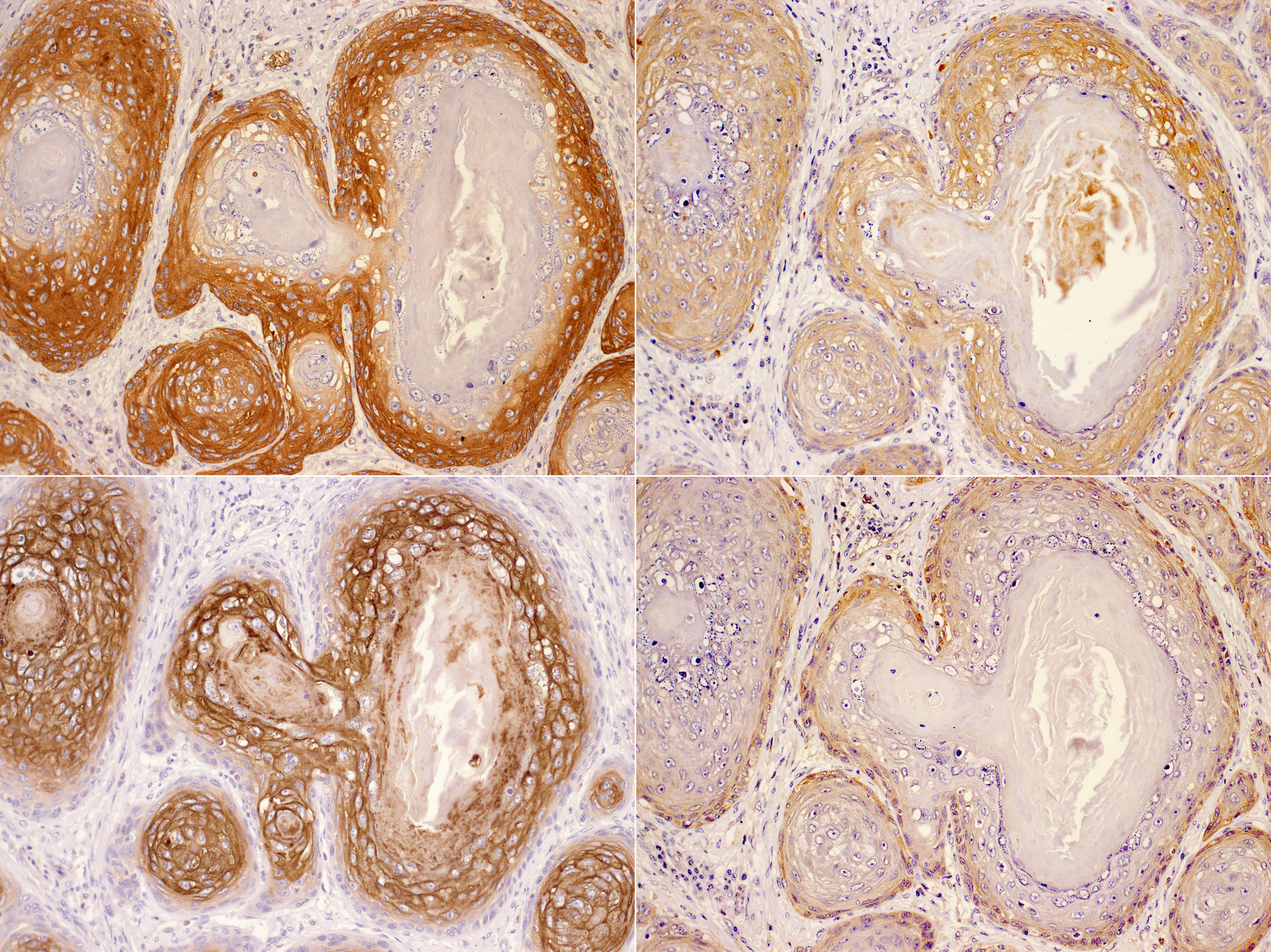

Supplement: Supplementary file 3 — Additional file 3: Figure S2: Staining patterns of GLUT-1 (upper left panel), CA IX (lower left panel), Hexokinase-2 (HK-2, upper right panel) and pyruvate kinase type M2 (PK-M2, lower right panel). GLUT-1 and CA IX both show a clearly demarcated and strong signal which is unequivocally restricted to a subtype of the cells present in the tissue slice. Conversely, HK-2 and PK-M2 staining is weak and more diffusely distributed throughout the tumor section. Images show the same subregion of the tumor but have not been registered using the described methodology. (JPEG 5 MB) [file 12885_2014_4944_MOESM3_ESM.jpeg]
